# Characteristics of the Gut Microbiome and Serum Metabolome in Patients with Functional Constipation

**DOI:** 10.3390/nu15071779

**Published:** 2023-04-06

**Authors:** Jialiang Wang, Linlin Wang, Qiangqing Yu, Nan Tang, Chunxia Mei, Hao Zhang, Gang Wang, Jian Lu, Wei Chen

**Affiliations:** 1 State Key Laboratory of Food Science and Technology, Jiangnan University, Wuxi 214122, China; 2 School of Food Science and Technology, Jiangnan University, Wuxi 214122, China; 3 National Engineering Research Center for Functional Food, Jiangnan University, Wuxi 214122, China; 4 (Yangzhou) Institute of Food Biotechnology, Jiangnan University, Yangzhou 225004, China; 5 Department of Gastroenterology, Wuxi No. 2 People’s Hospital (Jiangnan University Medical Center), Wuxi 214122, China

**Keywords:** functional constipation, gut microbiome, serum metabolome, arginine biosynthesis pathway, biomarker

## Abstract

Functional constipation (FC) is a gastrointestinal disorder with high incidence, and it seriously affects patients’ physical and mental health. Several studies have shown that the gut microbiome is associated with FC, but these studies have produced inconsistent findings, with few reflecting the relationship between the gut microbiome and metabolites. This study used 16S rRNA microbial genomics and non-target metabolome based on liquid chromatography–mass spectrometry to analyze the gut microbiota composition and serum metabolic profiles of 30 FC patients and 28 healthy individuals. We found that patients with FC and healthy individuals have different gut microbiota structures and serum metabolic profiles. FC patients had more Bacteroides and butyrate-producing bacteria (Roseburia, Faecaliberium, Butyriccoccus). The upstream products of host arginine biosynthesis (2-oxoglutaric acid, *L*-glutamic acid, *N*-acetylornithine, and *L*-ornithine) were significantly reduced in FC patients’ serum metabolites. In summary, our study describes the gut microbiome and serum metabolome of patients with functional constipation. It reveals that functional constipation may be associated with increased Bacteroidetes and downregulation of upstream products of host arginine biosynthesis, which may be potential markers for diagnosing functional constipation.

## 1. Introduction

Functional constipation is defined as constipation without organic causes and is diagnosed according to the Rome IV criteria [1]. The prevalence of FC is higher in women than in men [2]. The main symptoms of FC include labored bowel movements, decreased fecal water content, and decreased intestinal motility [3]. The etiology and pathophysiology of FC are largely unknown [4] and involve complex interactions of multiple factors, such as the central and peripheral nervous system, gut motility, intestinal barrier, and the balance of the gut microecology. The alteration of gut microecology is one possible cause, with studies finding that the gut microbiome is closely related to the diagnosis and treatment of FC [5]. A previous study showed that FC patients lack *Bacteroides*, *Coprococcus 3*, and *Roseburia* in their gut microbiota [6]. However, it has also been demonstrated that FC patients have elevated levels of *Bacteroides* and decreased levels of *Roseburia* and *Fusicatenibacter* [7]. Factors such as geographical location, age [8], the method of detection, and large individual variation may contribute to the inconsistent results. Another potential cause of the inconsistency is diagnostic accuracy. A study found that 89.5% of patients with irritable bowel syndrome–constipation (IBS-C) fulfill the criteria for FC, while 43.8% of patients with FC fulfill the criteria for IBS-C, and a subset of FC and IBS-C patients interconvert over time [9]. The diagnosis of constipation is based more on the judgment of physicians than on a pathological basis. However, in fact, IBS-C and FC patients may have distinct microbiomes. Different clinical symptoms of FC may be associated with different microbiomes. For example, the dominant microbiome in firmer stool samples is the Ruminococcaceae–Bacteroides enterotype [10].

Metabolites produced by gut microbes can potentially affect host health and contribute to the development of constipation. Short-chain fatty acids (SCFAs) and metabolites of dietary fiber fermentation by gut microbes are essential energy sources for colonic epithelial cells [11]. SCFAs can affect colonic motility by mediating glucagon-like peptide-1 (GLP-1) secretion [12] and regulating 5-hydroxytryptamine (5-HT) biosynthesis [13]. The gastrointestinal microbiota regulates the synthesis of bile acids (BAs), promotes the decoupling, dehydrogenation, and dihydroxylation of primary bile acids, and converts them into secondary bile acids [14]. BAs can affect intestinal peristalsis through the intestinal nervous system or endocrine system [15]. Clinical trials have shown that administration of chenodeoxycholic acid or ileal bile acid transporter inhibitors can shorten intestinal transit time and thus relieve constipation symptoms [16].

In this study, thirty patients diagnosed with functional constipation by Rome IV criteria were selected as the functional constipation (FC) group. At the same time, twenty-eight healthy individuals were chosen as the normal control (NC) group. All feces and serum specimens were analyzed. The compositions of the gut microbiome and serum metabolome were analyzed and compared by bioinformatics methods. By identifying the characteristic microbes and marker metabolites of FC by microbiomics and metabolomics approaches, this study hopes to provide new insights and references for future diagnosis and treatment of FC.

## 2. Materials and Methods

### 2.1. Participants and Sample Collection

Thirty FC patients and twenty-eight healthy people selected by Wuxi Second People’s Hospital were recruited for our study. All patients were diagnosed with functional constipation according to Rome IV criteria by medical doctors. All operation protocols were approved by the Ethics Committee of Wuxi Second People’s Hospital. All participants signed written informed consent forms before the collection of their fecal and blood samples. The exclusion criteria were as follows: (1) individuals with any underlying metabolic disease, (2) individuals who ingested alcohol within the two weeks before the sample collection, (3) individuals on medications, and (4) individuals who took antibiotics or probiotic formulations within one month before the sample collection. Healthy people were recruited according to the sex ratio and age requirement.

Fecal samples were transported to the laboratory within two hours of collection. Venous blood was drawn by professional nurses, and standard procedures were strictly followed to ensure sterility. Serums were collected by centrifugation. All samples in the laboratory were stored at −80 °C before being tested.

### 2.2. DNA Extraction and Sequencing from Fecal Samples

A Fast DNA Spin Kit for Feces (MP Biomedicals, Irvine, CA, USA) was used to extract total DNA from fecal samples. Universal primers (341F: 5′-CCTAYGGGRBGCASCAG-3′ and 806R: 5′-GGACTACNNGGGTATCTAAT-3′) were used to perform PCR amplification of the 16S rRNA V3–V4 region and the samples were barcoded.

The PCR products were separated on a 1.5% agarose gel and purified using the DNA Gel/PCR Purification Miniprep Kit BW-DC3511 (BIOMIGA, San Diego, CA, USA). The purified PCR products were quantified using the Qubit dsDNA Assay Kit (Life Technologies, Invitrogen, Waltham, CA, USA). In the final step, equal amounts of samples were mixed according to the concentration of PCR products, and an Illumina MiSeq PE300 was used for paired-end sequencing.

### 2.3. 16S rRNA Data Processing

Raw data of 16S rRNA sequence were processed by the QIIME2 software package. The amplicon sequence variants (ASVs) were rarefied to 10,000 according to the sampling depth. The α-diversity index was evaluated using the Shannon index, evenness index, Simpson index, and faith pd index, and the species richness was evaluated using the Chao1 index and observed_otus index. The α-diversity index was measured using Bray–Curtis distances.

### 2.4. LC/MS Non-Targeted Metabolomics Analysis

Precisely 100 mg of lyophilized fecal sample was weighed and dissolved by addition of 500 μL of MeOH-H2O (4:1, *v*/*v*). The mixture was homogenized and centrifuged at 15,000× *g* for 10 min. Subsequently, 400 μL of the mixture supernatant was collected and concentrated in vacuo until dry. The treated sample was redissolved with acetonitrile: water (1:1, *v*/*v*). The supernatant was centrifuged again at 15,000× *g* for 10 min, and the supernatant was used for ultra-high-performance liquid chromatography–tandem mass spectrometry (UHPLC-MS/MS).

The LC-MS system comprised an UltiMate 3000 UHPLC ultra-high-performance liquid chromatograph (Thermo Fisher Scientific, Waltham, MA, USA) and a Q-Exactive Plus high-resolution mass spectrometer (Thermo Fisher Scientific, Waltham, MA, USA). The system was used to detect metabolites in the host serum. The instruments’ operating conditions were set according to the method described by Zhu et al. [17]. Briefly, a Waters Acquity UPLC T3 column (2.1 × 100 mm, 1.8 µm) was used for gradient elution at an operating temperature of 30 °C. Mobile phases of (A) 0.1% formic acid–water and (B) acetonitrile were used for positive ion scanning modes; (A) 5 mmol/L ammonium acetate aqueous solution and (B) acetonitrile were used for negative ion scanning modes.

Mass raw data were processed by Compound Discovery 3.3 (Thermo Fisher Scientific, Waltham, MA, USA) for peak extraction, alignment, integration, and identification. Peaks with secondary mass spectra scores of over 0.8 were retained and matched by the databases of mzCloud and ChemSpider.

### 2.5. Statistical Analysis

R software (v 4.2.2) was used for statistical analysis and graphing. The characteristics of the FC and NC group were expressed as mean± SD or percentage. Student’s *t*-test and Fisher’s exact test were used to statistically analyze group differences in clinical indicators. The ANOSIM test, Wilcoxon rank-sum test, and linear discriminant analysis effect size (LEfSe) were used to explore the differences in microbiome. The Wilcoxon rank-sum test, fold change analysis, and orthogonal partial least-squares discriminant analysis (OPLS-DA) were used to process data and analyze metabolomics. The permutation test was performed by the SIMCA software (v 17.0.2). Spearman’s rank correlation coefficient was used to evaluate the correlation between microorganisms and metabolites. Pathway analysis of metabolite data was carried out via the MetaboAnalyst website (https://www.metaboanalyst.ca/, accessed on 20 November 2022). Network figures were obtained using the Gephi software (v 0.9.2).

## 3. Results

### 3.1. Basic Characteristics of the Study Cohort

This study recruited 30 constipation patients and 28 healthy individuals. Their clinical indicators are detailed in Table 1. The FC (42 ± 10.12) group and NC (46.43 ± 10.06) group had no significant age difference (*p* = 0.101). The FC (22.56 ± 2.81) group and NC (23.41 ± 2.84) group showed no significant difference in BMI (*p* = 0.127). The gender distributions of the two groups were also similar (*p* = 0.246). Defecation information was obtained from questionnaires. FC patients had lower defecation frequency (0.31 ± 0.13 vs. 0.70 ± 0.38) and a lower level of Bristol stool typing (1.93 ± 0.91 vs. 3.86 ± 1.41). Both were indications of defecation disorder in FC patients compared to NC individuals (*p* = 8.988 × 10^−6^, *p* = 1.826 × 10^−7^, respectively).

### 3.2. Changes in Intestinal Microbial Composition of Functional Constipation Patients

**Figure 1:**
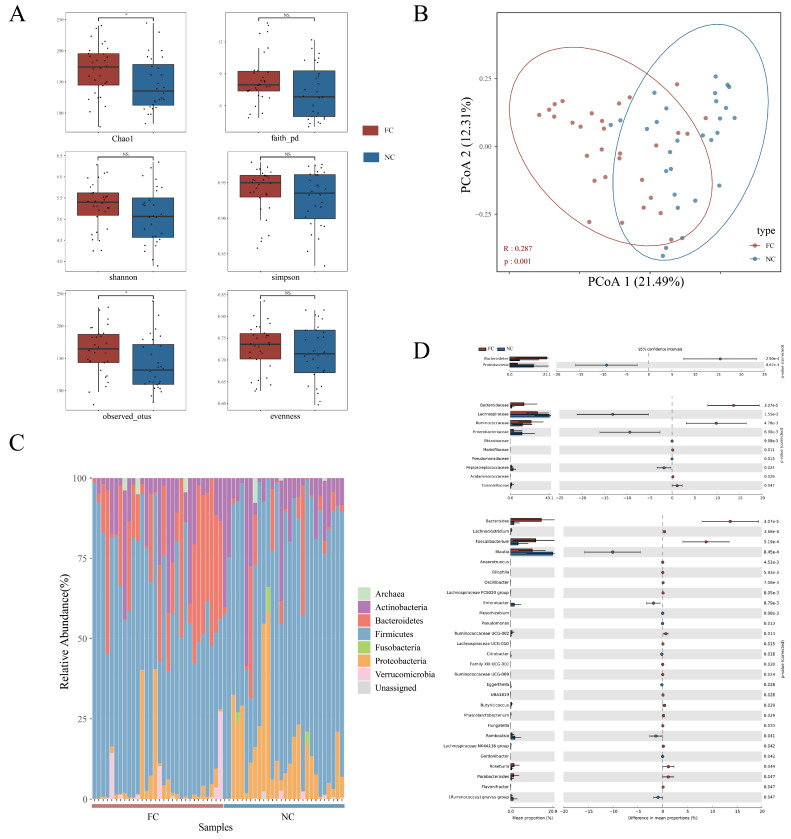
Intestinal microbiota community structure. (**A**) boxplot of six α-diversity indexes. (**B**) PCoA plot of β-diversity shows the differences between samples or groups. The horizontal and vertical axes are the two main coordinates with the greatest degree of explanation for the largest sample. (**C**) Stacked histogram shows the relative abundance of each individual’s gut microbes at the phylum level. (**D**) Differential microbes of the FC and NC groups at the phylum, family, and genus levels. * *p* < 0.05, NS *p* ≥ 0.05.

Several indexes were used to compare the α-diversities of FC and NC. The Chao1 index and observed OTUs of the FC group were higher, but the Shannon index, evenness index, Simpson index, and faith pd index of the two groups showed no significant difference (Figure 1A). Such results indicated that microbiota richness was higher in FC and that the microbiota evenness of the two groups had no difference.

A principal coordinate analysis (PCoA) showed that the community structures of intestinal microbiota in healthy people and FC patients were different (Figure 1B, *p* < 0.001, in the ANOSIM test). Stacked histograms illustrated the gut microbiota of each individual by phylum and Firmicutes, Bacteroides, Proteobacteria, and Actinobacteria had the highest relative abundance (Figure 1C).

We further compared the relative abundances of intestinal microbes in the two groups by phylum, family, and genus and identified two phyla, ten families, and twenty-eight genera (Figure 1D). At the phylum level, the abundance of *Bacteroides* in FC patients’ intestines significantly increased and *Proteobacteria* significantly decreased. There was no significant change in *Firmicutes*. At the family level, the abundances of *Bacteroidaceae* and *Ruminococcaceae* were higher in the FC group and *Lachnospiraceae* and *Enterobacteriaceae* were higher in the NC group. At the genus level, the main differential genera were *Bacteroides*, *Faecalibacterium*, *Ruminococcaceae UCG-002*, *Roseburia*, *Parabacteroides*, and *Lachnoclostridium* (higher in the FC group) and *Blautia*, *Enterobacter*, *Romboutsia*, and the *Ruminococcus gnavus group* (higher in the NC group).

### 3.3. Identification of Differences in Intestinal Microbiota

**Figure 2:**
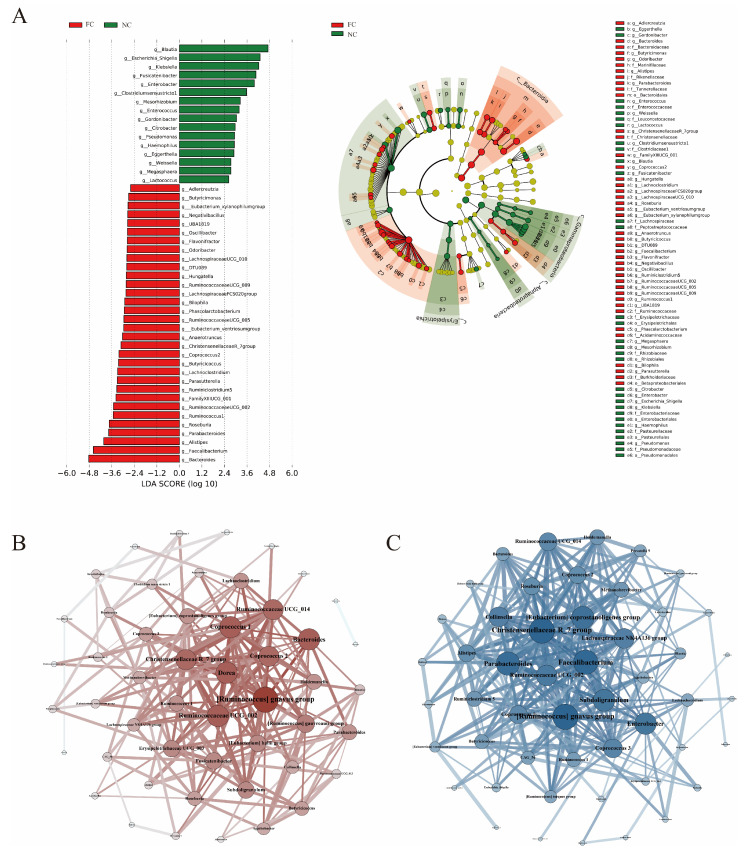
(**A**) LEfSe analysis for key genera selection. LDA score plot shows the differential genera. Higher LDA scores represent greater contribution of bacterial genera to the differences. Cladogram demonstrates differences at different levels. (**B**,**C**) Gut microbial network of the FC group and the NC group. The darker the dot’s color and larger the its size, the more related genera this genus has. A thicker line represents a stronger correlation.

LEfSe was used to identify the differential microbes (Figure 2A). In the FC group, 32 key genera were identified; in the NC group, the number was 16. *Blautia*, E*scherichia–Shigella*, *Klebsiella*, *Fusicatenibacter*, and *Enterobacter* were key genera in the NC group, and *Bacteroides*, *Faecalibacterium*, *Alistipes*, *Parabacteroides*, and *Roseburia* were key genera in the FC group.

In order to explore the connection between gut microbiota and FC symptoms, we associated intestinal microbiota with clinical indicators (Supplementary Figure S1). Forty-one genera were significantly correlated with the daily defecation frequency and Bristol stool typing. Eleven genera were only significantly correlated with age and BMI and may not be related to the development of constipation.

The dynamic balance of the intestinal ecosystem is maintained by interactions of intestinal microorganisms. Thus, we used Spearman’s correlation analysis to identify the core genera in the microecology (FDR < 0.05 and r > |0.3| for the top 50 most abundant genera) in FC and NC groups. We identified *Dorea*, *Ruminococcaceae UCG_002*, *Bacteroides*, and *Coprococcus 2* as the core genera in the FC group (Figure 2B) and the *[Eubacterium] coprostanoligenes group*, *Enterobacter*, *Parabacteroides*, and *Faecalibacterium* as the core genera in the NC group (Figure 2C). The (*Ruminococcus) gnavus group*, *Ruminococcaceae UCG_002,* and the *Christensenellaceae R_7 group* were the core genera in both the FC and NC groups.

### 3.4. Changes in Serum Metabolite Profile and Crucial Metabolites in Constipation Patients

The secondary products produced by the gut microbiota can enter the blood and have functional effects on the host’s physiology through the circulation of the whole body. Therefore, to further explore changes in gut microbe–host interactions, we detected serum metabolites based on non-targeted metabolomics using LC-MS. Principal component analysis (PCA) showed that the overall compositions of metabolites in the FC group and the NC group had a significant difference (*p* = 0.001, in the ANOSIM test), and QC points were clustered well, which indicated the instrument was stable (Supplementary Figure S2). A total of 115 positive and 349 negative ions were identified, and in the end, 292 metabolites were confirmed after merging and de-duplication.

**Figure 3:**
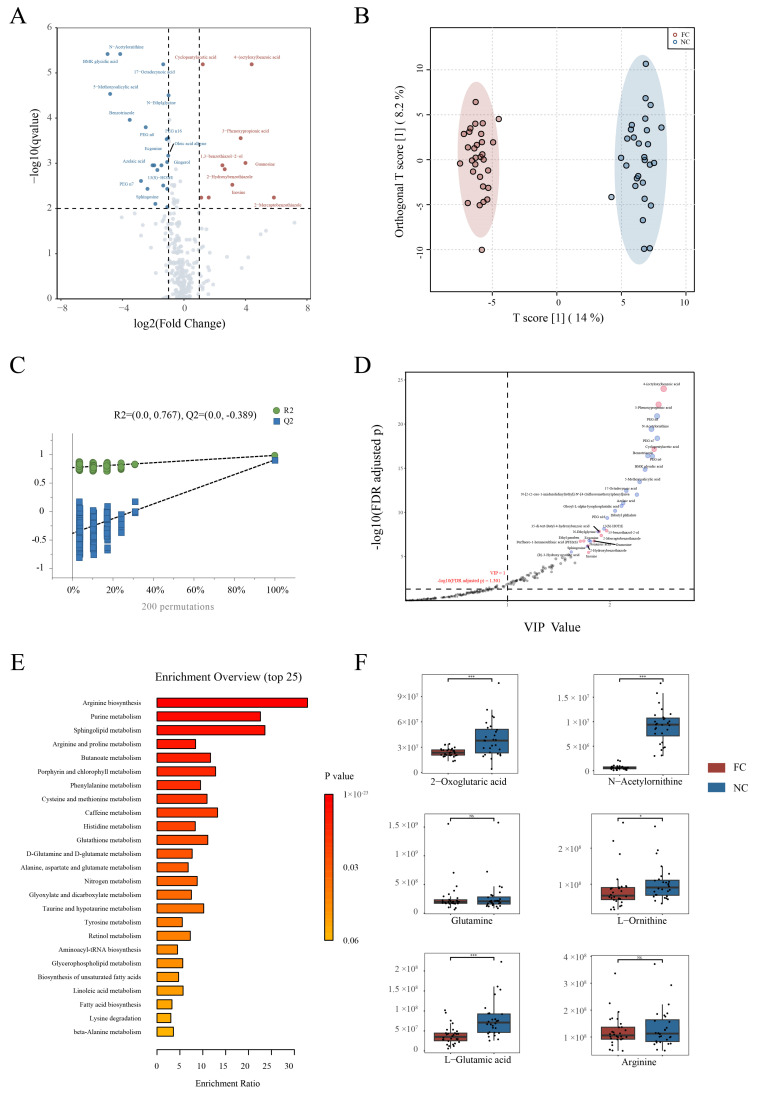
Serum metabolite structures. (**A**) Volcano plot shows differential metabolites with fold change > 1 and *p* < 0.01 between the two groups. The red points represent higher metabolite content in the FC group, while the blue points represent lower content. (**B**) OPLS-DA shows the differences in metabolites. The abscissa reflects the differences between the two groups, and the ordinate reflects the differences among samples within groups. (**C**) Permutation test based on OPLS-DA for validating the model’s reliability. (**D**) Point plot of VIP value and *p*-value. Red points represent higher metabolite content in the FC group, and blue points represent lower content. (**E**) Pathway enrichment bar plot. (**F**) Content of metabolites related to arginine biosynthesis pathway in the two groups. * *p* < 0.05, *** *p* < 0.001, NS *p* ≥ 0.05.

The volcano plot shows the differences in serum metabolism between the FC group and the NC group (FDR < 0.01, Wilcoxon rank-sum test, fold change > 2) (Figure 3A). Ten metabolites had higher levels in patients with FC than in the NC group and twenty-one had lower levels.

OPLS-DA was conducted to compare metabolic patterns based on non-targeted metabolomics. As is shown in the scatter plot (Figure 3B), the samples of the two groups could be easily distinguished, indicating that FC patients had a different metabolic profile from the NC group. The permutation test suggested that the model was credible and not overfitted (Figure 3C).

Variable importance of projection (VIP) values of the OPLS-DA model were used to screen differential metabolites. The differential metabolites of biological significance were screened according to the criteria of VIP value > 1 and FDR value < 0.05, and the larger the VIP value was, the greater the contribution of the variable to the grouping was. Ninety-six metabolites were selected by the OPLS-DA, and metabolites with the top thirty VIP values were annotated in the picture (Figure 3D). 4-(octyloxy) benzoic acid, 3-phenoxypropionic acid, cyclopentylacetic acid, 13-benzothiazol-2-ol, 2-mercaptobenzothiazole, and 3,5-di-tert-butyl-4-hydroxybenzoic acid in patients with constipation had higher abundance, while PEG n8, N-acetylornithine, PEG n7, benzotriazole, and PEG n6 had lower abundance.

To explore the relationship between blood metabolites and constipation, we correlated the metabolites with clinical indicators (Supplementary Figure S3). One hundred and forty-seven metabolites were significantly correlated with daily defecation times and Bristol stool typing. Sixty-six metabolites only had a significant correlation with age and BMI and may not be related to the development of constipation.

Metabolites were searched based on the KEGG human metabolic pathways database and used for pathway enrichment analysis (Figure 3E). The differential metabolic pathways were primarily involved in arginine biosynthesis, purine metabolism, sphingolipid metabolism, arginine and proline metabolism, butanoate metabolism, porphyrin and chlorophyll metabolism, phenylalanine metabolism, cysteine and methionine metabolism, caffeine metabolism, histidine metabolism, glutathione metabolism, *D*-glutamine and *D*-glutamate metabolism, alanine, aspartate and glutamate metabolism, nitrogen metabolism, glyoxylate and dicarboxylate metabolism, taurine and hypotaurine metabolism, tyrosine metabolism, retinol metabolism, aminoacyl-tRNA biosynthesis, glycerophospholipid metabolism, and biosynthesis of unsaturated fatty acids (*p* < 0.01, enrichment ratio was computed by hits / expected, where hits = observed hits; expected = expected hits).

We found that arginine biosynthesis seemed to have a strong association with the progression of constipation. Therefore, we compared the metabolites involved in this pathway, which included 2-oxoglutaric acid, *L*-glutamic acid, glutamine, arginine, *N*-acetylornithine, and L-ornithine, to find new biomarkers (Figure 3F). The results showed that 2-oxoglutaric acid, *L*-glutamic acid, *N*-acetylornithine, and *L*-ornithine were significantly lower in the FC group. Arginine and glutamine had no significant difference between the two groups.

### 3.5. Conjoint Analysis

**Figure 4:**
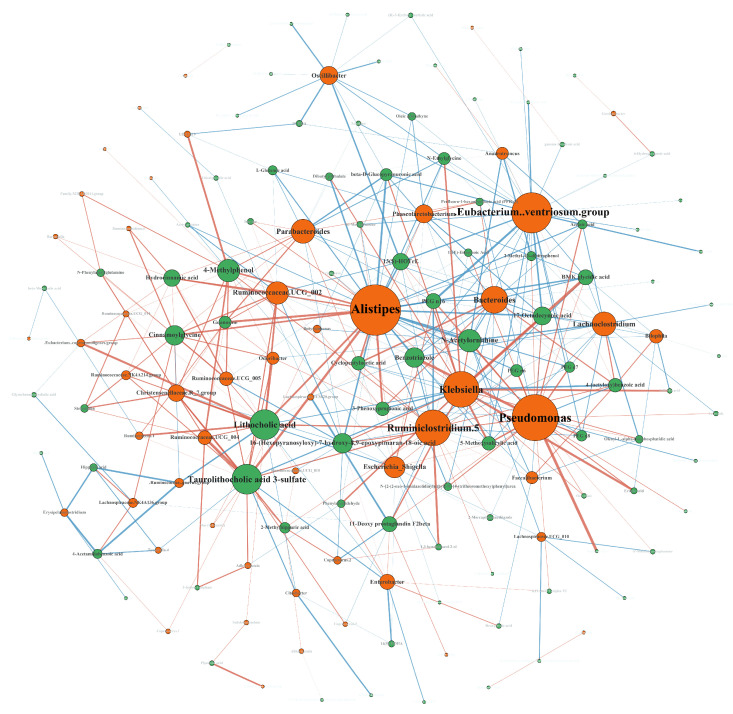
Correlation network of microbiome and metabolome. Correlation is represented by the connecting lines. A red connecting line represents a positive correlation between nodes, and a blue line represents a negative correlation. The size of a node is proportional to the number of lines it is connected to.

The direct or indirect secondary metabolites produced by intestinal microorganisms affect blood metabolites through the intestinal barrier. Correlation analysis was used to better characterize the relationship network of intestinal microorganisms and metabolites (Figure 4). *Alistipes*, *Ruminiclostridium 5*, *Klebsiella*, and the *[Eubacterium] ventriosum group* had the highest correlation with gut microbiota as they had the largest numbers and lithocholic acid. 4-methylphenol, taurolithocholic acid 3-sulfate, N-acetylornithine, and benzotriazole were the most correlated metabolites. These bacteria with more correlation had a greater influence on the change in metabolites.

## 4. Discussion

Our results indicate that patients with functional constipation have changes in their gut microbiota and serum metabolites. We combined genomics and metabolomics to search for occurrence mechanisms and potential biomarkers of FC.

Healthy controls were recruited by hospital physicians. All constipated patients were newly diagnosed by specialized physicians according to Rome IV criteria without treatment. All recruited individuals were not suffering from any other disease or had recently taken medication. Drugs and diets were important factors to consider when we screened the study subjects because they can greatly affect the human gut. Such factors include antibiotics, alcohol, yogurt, and drugs to treat constipation. We did not restrict the diet of the recruited individuals, but they were instructed to keep their usual diet until the samples were collected. They were also asked not to take anything that could have big impacts on their guts.

It is observed that there were differences in the composition and structure of intestinal microbiota in the FC group and the NC group. The relative abundance of the Bacteroidetes phylum, in which *Bacteroides* is the predominant genus, was significantly higher in the intestines of FC patients. Consistent with our findings, a cross-sectional study demonstrates that the relative abundance of Bacteroidetes significantly increases in constipated patients, and such an increase is positively correlated with the severity of FC [18]. Overgrowth of *Bacteroides* may lead to increased degradation of mucins’ protective and lubricating effects, resulting in thinner mucus layers and impaired intestinal barrier function [19]. As the core bacterium in the interaction network of FC, *Bacteroides* is positively correlated with a high-protein/low-fiber diet [20] and produces the inhibitory neurotransmitter γ-aminobutyric acid (GABA) [21], which may affect gastrointestinal motility depending on different GABA receptors [22]. Moreover, it is worth mentioning that genera identified as potential differential microbes, including *Alistipes*, *Butyricimonas*, and *Parabacteroides*, also belong to the phylum Bacteroidetes. In correlation analysis, *Bacteroides*, *Faecalibacterium*, *Alistipes*, and *Parabacteroides* were only correlated with lower Bristol stool typing and had no significant correlation with ages and BMI. E*scherichia–Shigella* and *Klebsiella* were only positively correlated with the daily defecation frequency and Bristol stool typing and are conditional pathogenic bacteria associated with diarrhea [23]. As members of Proteobacteria, they are regarded as harmful bacteria. However, in our study, their decrease in FC patients suggests that a certain abundance of *Escherichia–Shigella* and *Klebsiella* may benefit the balance of the intestinal microenvironment. *Alistipes* and *Parabacteroides* have been reported to increase in patients with slow transit constipation (STC), while *Klebsiella* is enriched in normal controls [24]. Such changes are consistent with our findings. In short, we consider the increase in Bacteroidetes (*Bacteroides*, *Alistipes*, *Butyricimonas*, *Parabacteroides*) and the decrease in *Blautia*, *Escherichia–Shigella,* and *Klebsiella* as one potential cause of functional constipation.

Our results also indicate that levels of butyrate-producing bacteria such as *Roseburia*, *Faecaliberium*, *Butyriccoccus*, and *Ruminococcaceae* [25] in the FC group are significantly higher than in the NC group. Some bacteria produce primary metabolites by degrading non-digestible carbohydrates, such as *Bifidobacteria*, *Lactobacillus*, *Bacteroides*, etc., and further cross-feed butyrate-producing bacteria to increase butyrate yield [26]. As one of the short-chain fatty acids, butyrate plays an important role in health maintenance and disease development [27]. Although many studies find that butyrate can benefit gastrointestinal motility and repair the intestinal barrier, different conclusions have also been reported [28]. These inconsistent results may be related to the concentration of butyrate. Unfortunately, we did not examine the levels of butyrate in feces, so there is no direct evidence of different butyrate concentrations in the FC and NC groups. However, our study illustrates that differences in butyrate-producing bacteria may contribute to constipation.

We used metabolic products for pathway enrichment analysis to find the mechanism of metabolic pathway changes in functional constipation. The arginine biosynthetic pathway had the highest enrichment ratio and the lowest *p*-value, meaning it was the most differential pathway between the two groups. A study indicated that genes involved in the arginine biosynthetic pathway in the gut microbiota were enriched in NC, not in FC, and they are associated with the development of constipation. A study showed that arginine content in the serum of constipation patients increased after receiving fecal microbiota transplantation (FMT) treatment [29]. Although no difference in arginine was found in our study, its upstream products, 2-oxoglutaric acid, *L*-glutamic acid, *N*-acetylornithine, and *L*-ornithine, were lower in the FC group than in the NC group. In the classical arginine pathway, *N*-acetylornithine is converted to ornithine by acetylornithine deacetylase or ornithine acetyltransferase and ornithine is converted to arginine by citrulline and argininosuccinate. Glutamate can be converted into *N*-acetylglutamate through catalysis, which then participates in arginine synthesis as a prerequisite for *N*-acetylornithine [30]. A study found that *L*-ornithine stimulated, but conversely, arginine suppressed, gastrointestinal motility after oral administration in mice [31]. Another cross-sectional study showed that levels of serum metabolites of *N*-acetyl-*L*-glutamic acid were significantly lower in constipated women of reproductive age than in healthy controls [32]. Interestingly, glutamate can be decarboxylated to GABA in addition to its conversion to N-acetylornithine, which is negatively associated with psychiatric disorders [33]. We speculate that this process may be related to intestinal microbiota.

In the correlation analysis, we explored the relationship between intestinal microbiota, apparent indicators, and serum metabolites. We added age and BMI to the correlation analysis to avoid their impacts on the results. For example, *Bacteroides* was associated with dryer and harder feces, and L-ornithine was associated with wetter and softer feces. Neither had a significant correlation with age, BMI, or defecation frequency. This means that their contribution to FC is credible. Glutamine was found to have no significant association with constipation symptoms, but it was negatively correlated with BMI. This finding suggests that BMI may be the influencing factor that masked the difference in glutamine between the FC and NC group. The correlations between gut microbiota and serum metabolites we found suggest that gut microbiota can influence systemic metabolic levels via metabolites. A study on Japanese patients with functional constipation found that the patients had a significantly lower proportion of genes responsible for D-arginine and D-ornithine metabolism in the colonic mucosal flora [33]. This result, to some extent, confirms our conjecture.

This study has potential limitations. First, although this study described the characteristics of the FC patients’ microbial composition, metabolite profiles, and their interactions, the generalizability of the results may have been influenced by diet, gender, region, and other factors. Larger cohorts examined with more precise methods such as metagenomics and targeted metabolomics are needed for validation. Second, we used a non-targeted metabolome to explore the changes in serum metabolites, which is not a comprehensive description. Therefore, future studies should expand the sample size and be conducted with fecal metabolomics. In summary, our findings provide a new reference for the gut microbiome and serum metabolome of FC patients and lay a foundation for further exploration of the etiology and treatment of functional constipation.

## 5. Conclusions

This study described the gut microbiome and serum metabolome of patients with functional constipation. Based on genomics, we found that FC patients have increased Bacteroidetes (*Bacteroides*, *Alistipes*, *Butyricimonas*, and *Parabacteroides*) and butyrate-producing bacteria (*Roseburia*, *Faecaliberium*, *Butyriccoccus,* and *Ruminococcaceae*) and decreased *Blautia*, *Escherichia–Shigella,* and *Klebsiella*. Based on metabolomics, we conclude that FC may be associated with downregulation of the pathway’s upstream products (2-oxoglutaric acid, *L*-glutamic acid, *N*-acetylornithine, and *L*-ornithine) in the serum of the host associated with arginine biosynthesis. The changes in gut microbes and serum metabolites may be potential markers for FC diagnosis and treatment.

## Figures and Tables

**Table 1:** Characteristics between patients of the FC group and the NC group.

Variable		Constipation (*n* = 30)	Health (*n* = 28)	*p*-Value
Age, Mean ± SD		42 ± 10.12	46.43 ± 10.06	0.101
Sex, *n* (%)	Female	28(93.3)	23(82.1)	0.246
	Male	2(6.7)	5(17.9)	
BMI, Mean ± SD		22.56 ± 2.81	23.41 ± 2.84	0.127
Defecation frequency, Mean ± SD (times/day)		0.31 ± 0.13	0.70 ± 0.38	8.988 × 10^−6^
Bristol stool typing, Mean ± SD		1.93 ± 0.91	3.86 ± 1.41	1.826 × 10^−7^

## Data Availability

The datasets generated and analyzed during the current study are available from the corresponding author upon reasonable request.

## Supplementary Materials

The following supporting information can be downloaded at: https://www.mdpi.com/article/10.3390/nu15071779/s1, Figure S1: Heat map of correlation between clinical indicators and bacteria; Figure S2: PCA diagram of LC-MS results; Figure S3: Heat map of correlation between clinical indicators and serum metabolites.
