# Supplementary figures and images for: Characteristics of the Gut Microbiome and Serum Metabolome in Patients with Functional Constipation
Source: Nutrients. 2023 Apr 6;15(7):1779. doi: 10.3390/nu15071779 (PMC10097253; doi:10.3390/nu15071779)

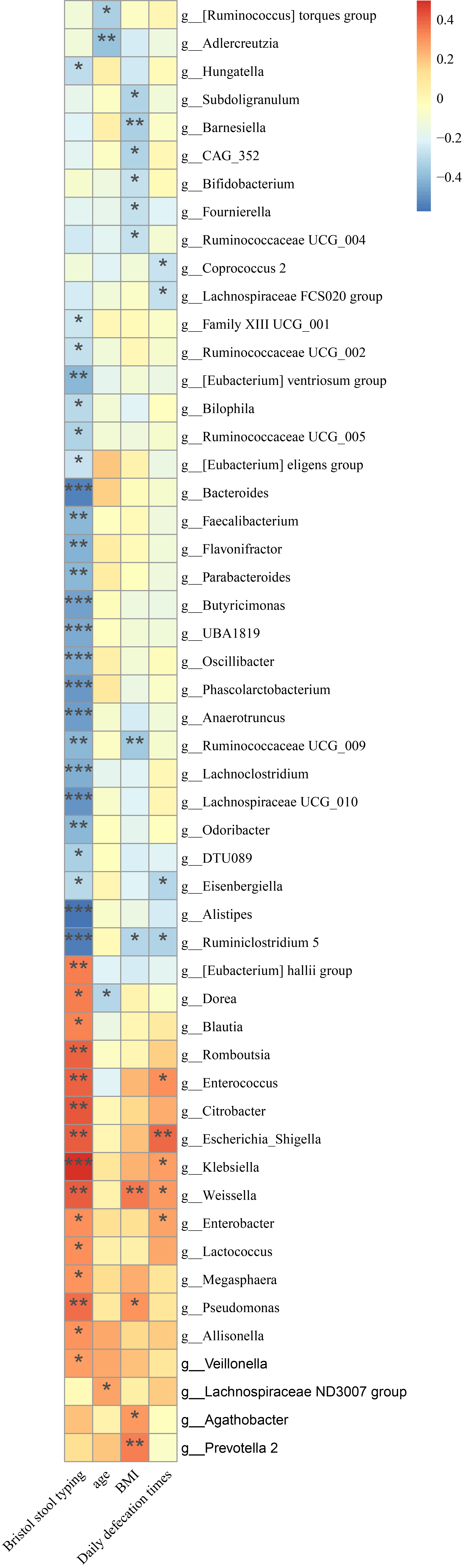

Supplement: Supplementary file 1 [file nutrients-15-01779-s001.zip › Figure S1.jpg]

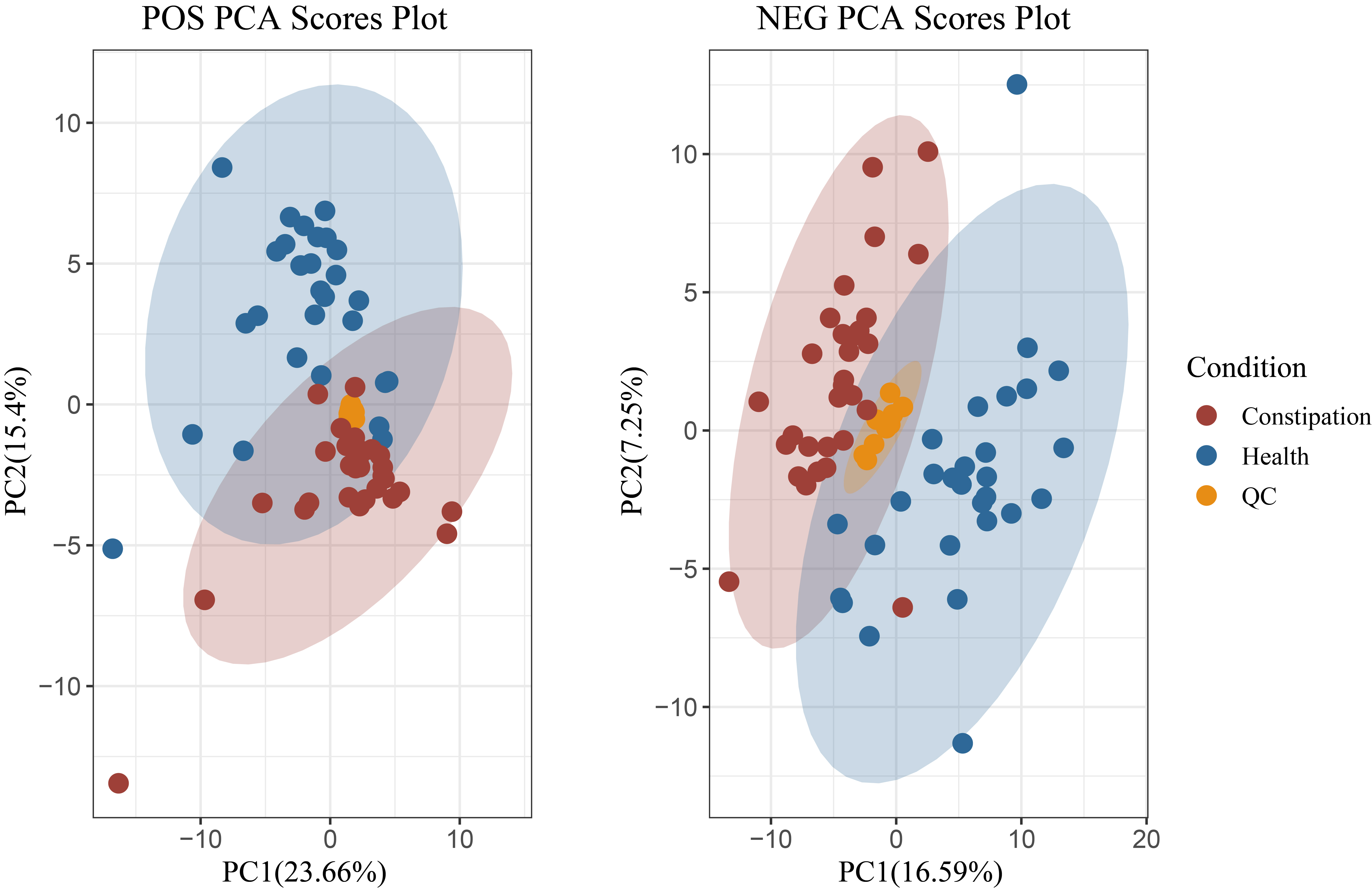

Supplement: Supplementary file 1 [file nutrients-15-01779-s001.zip › Figure S2.jpg]
